# Overexpression of centromere protein K (CENPK) gene in Differentiated Thyroid Carcinoma promote cell Proliferation and Migration

**DOI:** 10.1080/21655979.2021.1911533

**Published:** 2021-04-27

**Authors:** Qizhi Li, Jiang Liang, Shuai Zhang, Ning an, Lingfeng Xu, Changhong Ye

**Affiliations:** a Department of Head and Neck Surgery, Hubei Cancer Hospital, Tongji Medical College, Huazhong University of Science and Technology,Wuhan, China; b Department of Gynecology, Maternal and Child Health Hospital of Hubei Province, Tongji Medical College, Huazhong University of Science and Technology, Wuhan, China

**Keywords:** Centromere protein k, thyroid carcinoma, carcinogenesis

## Abstract

Differentiated thyroid carcinoma (DTC) is one of the most common malignant tumors. Increasing evidence indicates that centromere protein K(CENPK) may play a key role in promoting carcinogenesis. The expression, biological functions, and clinical significance of CENPK in DTC are still unclear. The CENPK expression in the DTC specimen was confirmed using quantitative real-time PCR and Western blot. The expression of CENPK was silenced and promoted by lentivirus-mediated transfection with shRNA sequences or CENPK plasmid targeting CENPK in TPC1 and FTC-133 cells, respectively. Colony formation, Cell Counting Kit-8 (CCK-8), Transwell invasion, and scratch assays were performed to assess the malignant biological properties of FTC-133 and TPC1 cells. Tumorigenicity assay was performed using C57BL/6 mice to explore the influence of CENPK on the growth of TPC1. The present work suggested that the expression of CENPK remarkably increased in follicular thyroid cancer and papillary thyroid cancer  tissue samples at the mRNA level. Immunohistochemical staining also showed consistent results at the protein level. In addition, CENPK mRNA expression level showed great value in diagnosis of DTC. Knockdown of CENPK significantly inhibited the invasion and migration of TPC1 and FTC-133 cells. In contrast, CENPK overexpression promoted invasion and migration of TPC1 and FTC-133 cells. Knockdown and overexpression of CENPK showed consistent effect on DTC tumor growth and expression of Ki-67 invivo. Our results indicated that CENPK was evidently upregulated in DTC. Knocking down CENPK suppressed TPC1 cell proliferation, invasion and migration. Targeting the CENPK may be anovel therapeutic method for DTC.

## Graphical abstract

**Figure:**
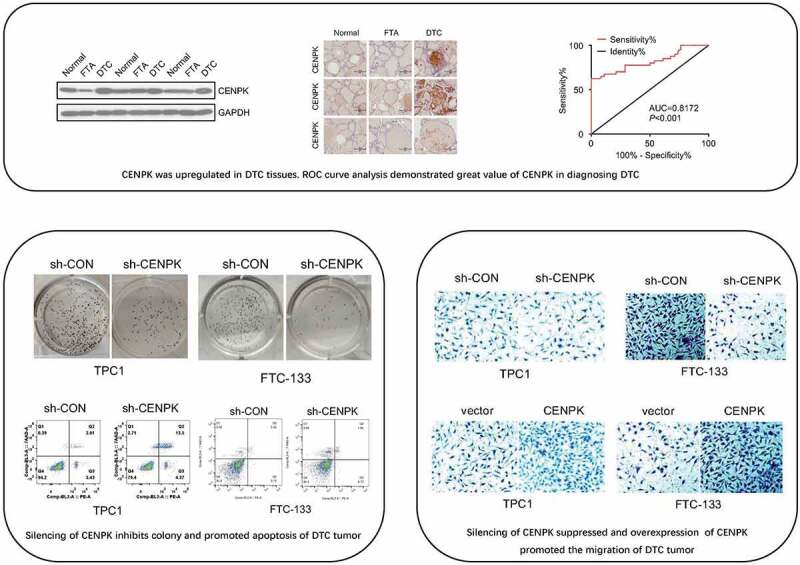

## Introduction

Differentiated thyroid cancer (DTC) represents a frequently seen malignant tumor mainly involving women, with a morbidity of 2 to 8 per 100,000 women [1]. According to histology, thyroid cancer can be divided into undifferentiated or differentiated subtypes. In addition, DTC can be further divided into follicular thyroid cancer (FTC) and papillary thyroid cancer (PTC), accounting for 75% and 16% of all thyroid cancers, respectively [2]. Over 95% of the patients with DTC can live more than 5 years with suitable and timely treatment [3]. Nevertheless, there is still a small proportion (5–20%) of patients showing aggressive behavior, also with poor prognosis [4,5]. Therefore, it is of critical importance to identify the novel diagnostic markers and therapeutic targets for DTC patients so as to improve their quality of life and survival rate.

Cumulative evidence indicates that dysfunction or dysregulation of kinetochore may cause aneuploidy and promote carcinogenesis [6]. Kinetochore, one of the protein structures in chromatids, exerts an important role in segregation of chromosomes in the processes of meiosis and mitosis [7]. There are over 80 distinct proteins in kinetochore, many of which, including CENP (centromere protein), are conserved among species [8]. Elevated expression of CENP-A is detected in ovarian cancer, CENP-E up-regulation is found in breast cancer (BC), whereas increased CENP-F expression is measured in prostate cancer (PCa) [9–11]. CENP-A is a recognized component of kinetochore involved in several human malignancies [12,13]. Previous research demonstrated that, in human cancer genome, the over-accumulation of CENP-A possibly alters chromosomal effect fragility and chromatin fiber state, and CENP-A overexpression predicts poor prognosis [13,14]. CENPK is another type of CENP, localized at the inner plate of kinetochore. It plays an essential role in facilitating efficient assembly of CENP-A with other components and maintaining normal function of kinetochore [15,16]. Several studies have shown that CENPK may be essential in the pathogenesis of human tumors, such as bladder cancer, liver cancer, and ovarian cancer [17–19]. However, the expression, biological functions, and clinical significance of CENPK in DTC are still unclear.

The present study was designed to explore the CENPK function and its underlying clinicopathological mechanism during DTC occurrence. CENPK mRNA expression was measured within PTC, FTC and FTA (follicular thyroid adenoma) specimens and explored the roles of CENPK in DTC carcinogenesis, proliferation, and migration both in vivo and in vitro.

## Methods

### Patients, tissues specimens, and datasets

DTC and adjacent normal thyroid tissue samples utilized in the present work were obtained from thyroid tissues in 30 FTA, 30 PTC, and 30 FTC surgical cases at the Hubei Cancer Hospital between January 2016 and December 2019, and clinical data were also obtained. None of the included patients were treated with radiotherapy and/or chemotherapy before surgery. Each tissue sample collected was subjected to immediate liquid nitrogen freezing and preservation under −80 °C after surgical resection. The diagnosis was confirmed by a postoperative pathological examination.

The Gene Expression Omnibus (GEO) database (www-ncbi-nlm-nih-gov.webvpn.cams.cn/geo) was adopted to obtain the gene expression datasets GSE 83,520 [20] and GSE82208 [21]. A total of 12 PTC tissue samples and 12 normal tissue samples in the GSE 83,520 dataset, and 25 FTA tissue samples and 27 FTC tissue samples in the GSE82208 datasets were analyzed for CENPK expression using R software and Bioconductor packages.

## Cell lines and culture conditions

Human PTC cell (TPC1) and FTC cell (FTC-133) were purchased from Shanghai Biochip Company Ltd. (Shanghai, China). TPC1 and FTC-133 cell lines were cultivated in RPMI 1640 (GIBCO) that contained 10% fetal bovine serum (FBS, GIBCO), 1 × MEM nonessential amino acids, and 1 × sodium pyruvate, and incubated under 5% CO_2_ and 37 °C conditions.

## Plasmids and cell transfection

Lentiviral plasmids that contained shRNA sequences used to suppress expression targeting CENPK (sh-CENPK) or negative control shRNA (sh-CON) were prepared by Genechem, and the CENPK expressing plasmid and vector plasmid were purchased from Genechem. After reaching 30% confluence, lentiviral plasmids were used to transfect TPC1 and FTC-133 cells cultured within the 6-well plates. Afterward, lentivirus-containing transduction-enhancing solution (polybrene, 50 µg/mL; multiplicity of infection, 20) was used to replace the original medium. At 12 h later, complete medium was used to replace the medium with culture cells for additional 72 h. Thereafter, 1 µg/mL puromycin was used to select cells, and cells were collected to carry out subsequent analyses.

## RNA extraction and Real-time quantitative PCR (RT-qPCR)

The RNAiso Plus reagent (TaKaRa, Dalian, China) was utilized to isolate total RNA from frozen tissue samples or TPC1 and FTC-133 cell lines. We used PrimeScript 1^st^ strand cDNA Synthesis Kit (Takara Bio, Otsu, Japan) to prepare first-strand cDNA following specific instructions. SYBR Premix EX Taq^TM^ II kit (TaKaRa) together with RT primers was adopted in RT-qPCR. The CENPK primers used in this experiment were as follows: 5′-GTTTGTGACGCTGTGATGGTCT-3′ (forward) and 5′-ACGCTTGAGGATGCAAGATGT-3′ (reverse). The present study used β-actin as a quantitative control to standardize expression of mRNA, and its primer sequences were shown below, 5′-GGACTTCGAGCAAGAGATGG-3′ (forward) and 5′-AGCACTGTGTTGGCGTACAG-3′ (reverse). β-actin was 234 bp in length, while CENPK was 121 bp in length. Genechem designed, produced, and validated all primers. GAPDH was used as the internal reference.

## Western blotting WB analysis

WB analysis was performed to measure the CENPK level within isolated cells. Cells were lysed by the lysis buffer (consisting of 25 mM Tris-HCL (pH 7.6), 1% sodium deoxycholate, 150 mM sodium chloride, 0.1% SDS and 1% NP-40) for 30 min at 0 °C. After sonication for 10 sec, the lysed cells were subjected to 20 min of centrifugation at 15,000 × g and 4 °C. Total protein was obtained from lysed cells using RIPA buffer (TaKaRa, Dalian, China). The buffer was added with protease inhibitor. The protein level was then quantified using a bicinchoninic acid protein assay kit (TaKaRa, Dalian, China). Subsequently, 30 µg protein was separated by 10% SDS-PAGE and added into each well of the vertical electrophoresis tank. Next, the products were transferred onto the PVDF membranes, followed by 2 h of blocking by 5% skimmed milk powder under ambient temperature and overnight incubation under 4 °C, using either the anti-CENPK polyclonal antibody (26,208–1-aP) (cat. no. 26,208–1-AP; 1:1000; Proteintech, Chicago, IL, USA) or control anti-β-actin antibody (cat. No. 60,008–1-ig; 1:10,000; Proteintech, Chicago, IL, USA) Then, the membranes were rinsed three times using 0.1% TBS-Tween-20 (5 min per time), followed by 2 h of incubation using horseradish peroxidase(HRP)–labeled goat anti-mouse/rabbit IgG (1:5,000; cat. nos. ab205718 and ab205719; Abcam) under ambient temperature. Later, TBS was used to rinse the membranes, and then blots were developed by ECL with chemiluminescence kit (Beijing Solarbio Science & Technology Co., Ltd.). An ECL detection system (Shanghai Biochip Company Ltd.) was used to detect the protein bands.

## Immunohistochemistry (IHC)

IHC was performed for evaluating the protein level of CENPK. To this end, the samples were embedded in paraffin, fixed with formalin, and sliced into the 3-μm sections. Xylene was used to dewax, and the sections were hydrated with alcohol. After washing with PBS, the sections were subjected to high-pressure stream for 30 min of antigen retrieval within the citrate buffer (pH 6.0). Thereafter, cells were incubated with rabbit anti-CENPK monoclonal primary antibody (1:400, Abcam, Cambridge, UK) overnight under 4°C, followed by incubation using the secondary antibody (SP9000; goat anti-mouse IgG; OriGene Technologies, Inc., Beijing, China), the sections were stained with DAB (OriGene Technologies, Inc., Beijing, China) before incubating. The nuclei were stained with Mayer hematoxylin for 30 s.

## CCK-8 assay

Transfected TPC1 cells and FTC-133 cells (3,000/well) were inoculated into the 120-well plates. CCK-8 (EMD Millipore, Billerica, MA, USA) assay was conducted to evaluate cell proliferation based on the protocols for manufacturers every day for 4 days.

## Colony formation assay

Transfected TPC1 cells and FTC-133 cells (600/well) were seeded into the 12-well plates. After being incubated for 14 days, the colonies were then rinsed by PBS and fixed for 20 minutes with 95% ethanol solution, followed by 20 min of 0.4% crystal violet staining. At last, the microscope was used to count the formed colonies.

## Wound healing assay and invasion assay

The FTC-133 and TPC1 cell migration was evaluated by wound healing assay. After cultured in 6-well plates in a tight cell monolayer, TPC1 cells and FTC-133 cells were wounded with 100 μL plastic pipette tips. Photos of the wound front were taken by microscopic at 200× magnification. Then the migrating distance of cells was measured. Invasion of TPC1 cells and FTC-133 cells were detected using the Transwell chambers coated with Matrigel (Yiyuan Biotechnologies, Guangzhou, China) for 5 h under 5% CO_2_ and 37 °C conditions. Altogether 5 × 10^4^ cells were inoculated into the upper chamber onto the Matrigel-coated membrane. Cells were allowed to migrate toward the lower chamber in which contained 500 ml complete medium (RPMI medium with 1% penicillin/streptomycin and 5% FBS). Incubated at 37 °C for 24 hours, then 4% paraformaldehyde was utilized to fix cells for 20 min, followed by 20 min of 0.4% crystal violet staining. We counted the invaded cells in five different fields at 200× magnification.

## Tumorigenicity in nude mice

The athymic male nude mice aged 4–6 weeks were given subcutaneous injection of TPC1-shNC, FTC-133-shNC, TPC1-shCENPK and FC-133-shCENPK cells via the right back to generate xenograft tumors. We determined the tumor volume through the formula shown below: tumor volume = (length×width [2]/2). According to the guidelines for the care of animals, CO_2_ inhalation was applied in mice after 24 days for euthanasia; then, the tumors were collected and weighed using electronic weigher. Moreover, tumor growth was evaluated by the bioluminescence imaging.

## Statistical analysis

All experiments were performed in triplicate, based on which the statistical analysis was conducted. The continuous data were expressed as the mean ± SD. Student’s t-tests (two-tailed) were used to detect significant differences of two groups via SPSS22.0 (Chicago, IL, USA). A difference of P < 0.05 suggested statistical significance.

## Ethics statement

The Hubei Cancer Hospital Ethical Committee (Wuhan, China) approved the present work. All patients provided informed consent for using the necessary biological material. Male C57BL/6 mice (8–10 weeks old) were bought from Shandong University Animal Ethical Committee (Jinan, China). All animal experiments were approved by The Animal Care and Use Committee of Tongji Medical College (Wuhan, China) approved all animal experiments performed following the guidelines for the care and use of laboratory animals released by the National Institutes of Health.

## Results

The present work examined the CENPK function and possible clinicopathological mechanism underlying DTC occurrence. We detected the mRNA and protein levels of CENPK in PTC specimens, FTC specimens, adjacent normal thyroid tissues, and FTA specimens and investigated the roles of CENPK in DTC progression in vitro and in vivo.

## CENPK is upregulated in DTC tissues

**Figure 1.**
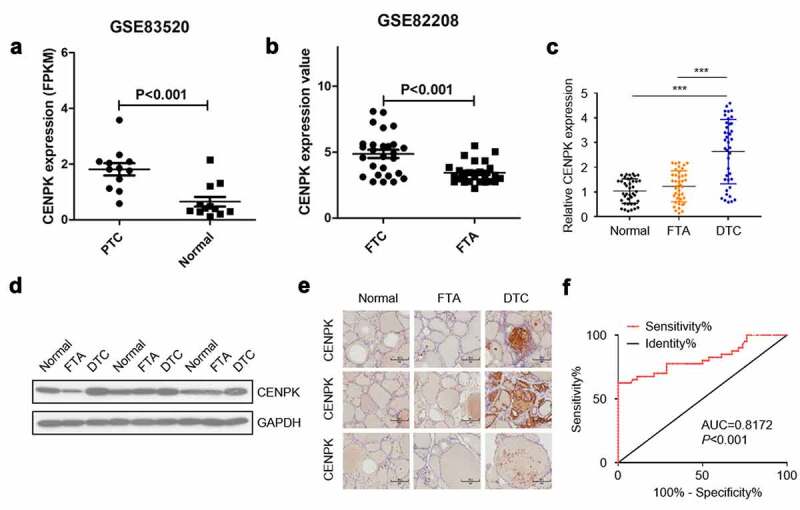
CENPK is upregulated in DTC tissues

**Notes**: (A) Expression of CENPK was significantly upregulated in PTC tissues than that in 12 normal tissue (P < 0.001). (B) Expression of CENPK was significantly upregulated in 27 FTC tissue than that in 25 FTA tissue (P < 0.001. (C) Relative mRNA level of CENPK was significantly upregulated in DTC tissues by qRT-PCR. (D) Protein level of CENPK in DTC tissues was significantly higher than that in normal thyroid tissues as detected by western blotting. (E) Immunohistochemistry staining showed that CENPK was negative in FTA and normal tissues but was highly expressed in DTC tissue. (F) ROC curve analysis for the predictive power of CENPK using qRT-PCR expression dataset. *P < 0.05, **P < 0.01, ***P < 0.001.

The expression of CENPK in the Gene Expression Omnibus database was determined for assessing the possible CENPK function in FTC and PTC, respectively. We observed that the expression of CENPK markedly increased within FTC and PTC tissue samples in comparison with the FTA and matched non-carcinoma thyroid tissues, respectively (Figure 1a-B). Relative mRNA level and protein level of CENPK were significantly upregulated in DTC tissues detected by qRT-PCR and western blotting (Figure 1c-D). Immunohistochemistry staining showed that CENPK was overexpressed in the DTC tissue but was negative in both FTA and normal tissues (Figure 1e). CENPK mRNA expression was used as datasets for ROC curve analysis. Using a cutoff probability of 50%, we obtained sensitivity of 74% and specificity of 88%. The calculated area under the ROC curve was 0.81, indicating a great diagnosis value of CENPK in DTC (figure 1f). Overall, these results showed that the mRNA and protein expression of CENPK increased within DTC tissue samples compared with adjacent normal tissues, indicating the possible role of CENPK in promoting DTC development.

## Knockdown of CENPK inhibits DTC cell proliferation and colony

**Figure 2.**
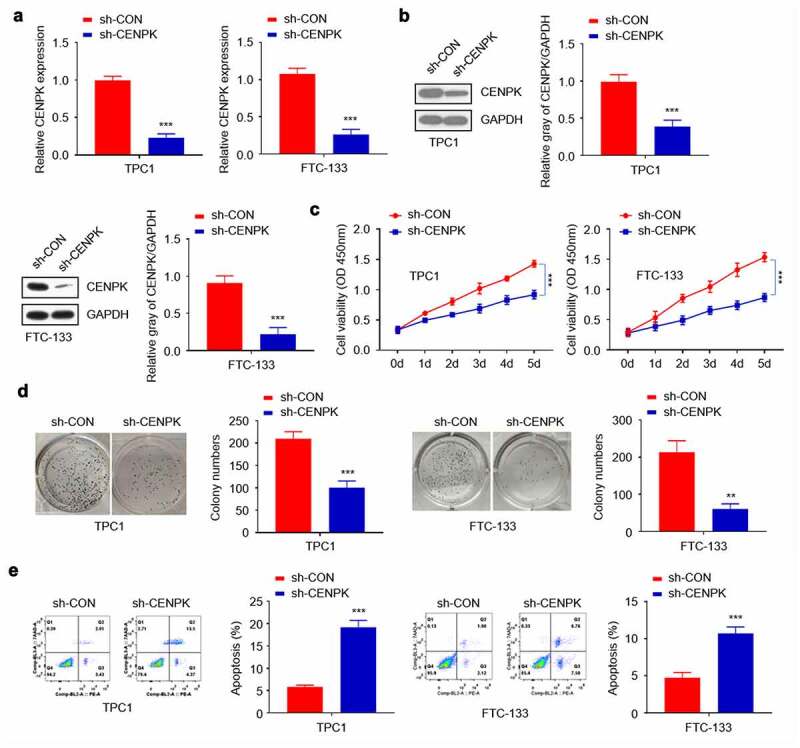
Silencing of CENPK inhibits DTC cell proliferation and colony

**Notes**: (A) Relative mRNA level of CENPK was significantly suppressed in TPC1 cells when transfected with sh-CENPK-encoding lentivirus. (B) The protein expression of CENPK was significantly suppressed in both TPC1 cells and FTC-133 cells when transfected with sh-CENPK-encoding lentivirus as shown by Western blotting. (C) The downregulation of CENPK evidently suppressed cell viability as shown by CCK-8 assay. (D) Silencing of CENPK reduced the colony number of both TPC1 cells and FTC-133 as shown by colony formation assays. (E) Knockdown of CENPK promoted apoptosis of TPC1 cells as shown by Annexin V Apoptosis Detection Kit. *P < 0.05, **P < 0.01, ***P < 0.001.

To further explore CENPK’s biological role in DTC occurrence, the endogenous CENPK levels within both TPC1 cells and FTC-133 cells were down-regulated using lentivirus-mediated shRNA. Transfected with sh-CENPK-encoding lentivirus and then CENPK mRNA and protein levels in both TPC1 cells and FTC-133 cells were inhibited (Figure 2a-B). The influence of CENPK silencing on the proliferation of both TPC1 cells and FTC-133 cells was subsequently investigated through clone forming and CCK-8 assays. According to our findings, the downregulated CENPK evidently suppressed cell viability and the colony number verified in both TPC1 cells and FTC-133 cell (Figure 2c–D). Apoptosis was analyzed by Annexin V Apoptosis Detection Kit. We found that the knockdown of CENPK promoted apoptosis of both TPC1 cells and FTC-133 cells (Figure 2e).

## Overexpression of CENPK promote DTC cell proliferation and colony

**Figure 3.**
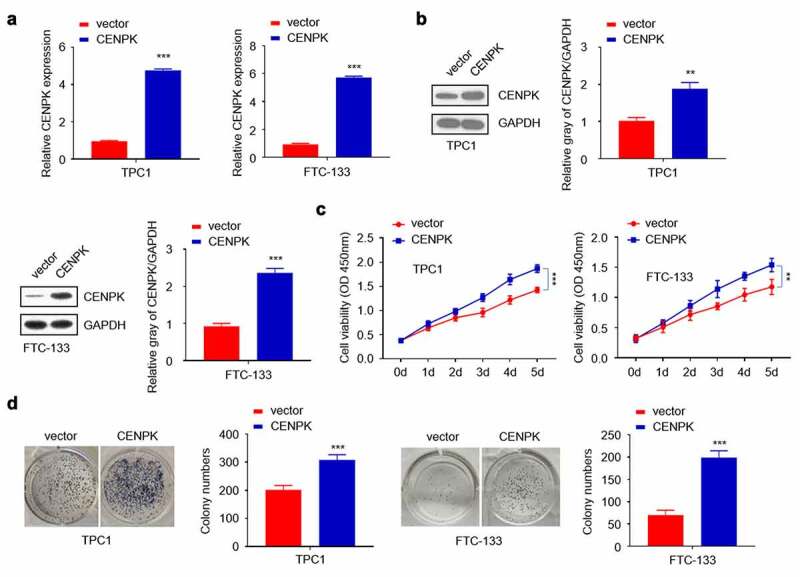
Overexpression of CENPK promotes DTC cell proliferation and colony

**Notes**: (A) Expression of CENPK was significantly elevated in both TPC1 cells and FTC-133 cells transfected with CENPK plasmid. (B) The mRNA and protein expression of CENPK was significantly elevated in both TPC1 cells and FTC-133 cells transfected with CENPK plasmid. (C) The upregulation of CENPK evidently promoted cell viability as shown by CCK-8 assay. (D) Overexpression of CENPK promoted the colony number verified by both TPC1 cells and FTC-133 cells as shown by colony formation assays. *P < 0.05, **P < 0.01, ***P < 0.001.

We used CENPK plasmid to promote the endogenous expression of CENPK in both TPC1 cells and FTC-133 cells. After transfection with CENPK, CENPK mRNA and protein levels markedly increased within both TPC1 cells and FTC-133 cells (Figure 3a-B). The influence of CENPK silencing on proliferation of both TPC1 cells and FTC-133 cells was subsequently investigated. Based on clone forming and CCK-8 assays, CENPK up-regulation markedly enhanced the number of formed colonies and cell viability in both TPC1 cells and FTC-133 cells (Figure 3c–D).

## Influence of knockdown and overexpression of CENPK on DTC tumor migration, and invasion in vitro

**Figure 4.**
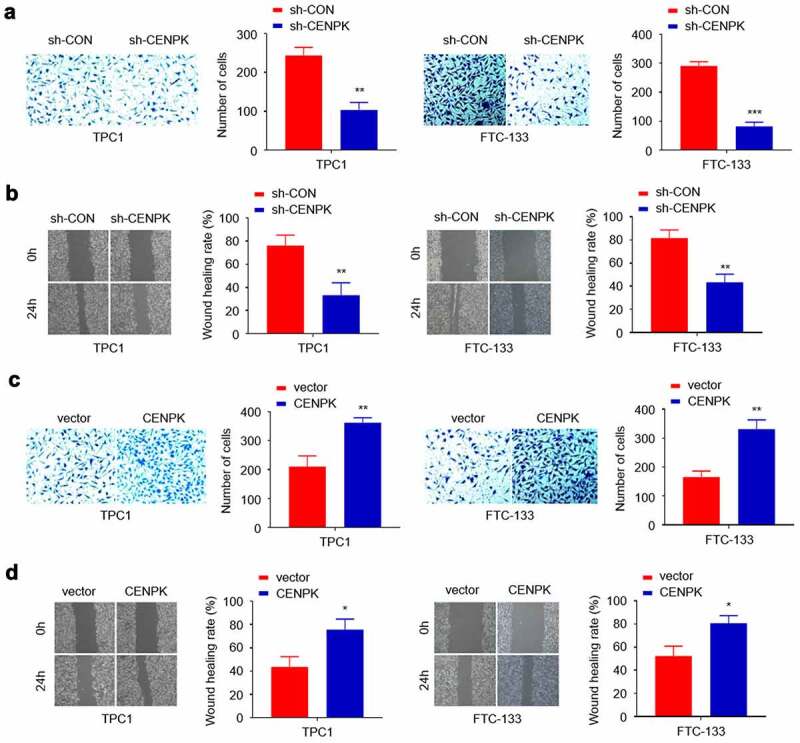
Influence of knockdown and overexpression of CENPK on DTC tumor migration, and invasion in vitro

We conducted scratch and Transwell assays for assessing the CENPK role in cell invasion and migration. Transfected with the sh-CENPK-encoding lentivirus, FTC-133 and TPC1 cell invasion and migration were then significantly decreased compared with the sh-CON group (Figure 4a–B). When transfected with the CENPK plasmid, the upregulated CENPK promoted those of TPC1 cells compared with the vector group (Figure 4c–D). Overall, the above findings suggested the active involvement of CENPK in migration and invasion of both TPC1 cells and FTC-133 cells.

**Notes**:(A) Silencing of CENPK suppressed the migration of both TPC1 cells and FTC-133 cells as detected by wound healing assay. (B) Silencing of CENPK suppressed the invasion of both TPC1 cells and FTC-133 cells as detected by transwell assays. (C) Overexpression of CENPK promoted migration of both TPC1 cells and FTC-133 cells. (D) Over expression of CENPK promoted invasion of TPC1 cells. *P < 0.05, **P < 0.01, ***P < 0.001.

## Influence of knockdown and overexpression of CENPK suppresses DTC tumor growth in vivo

**Figure 5.**
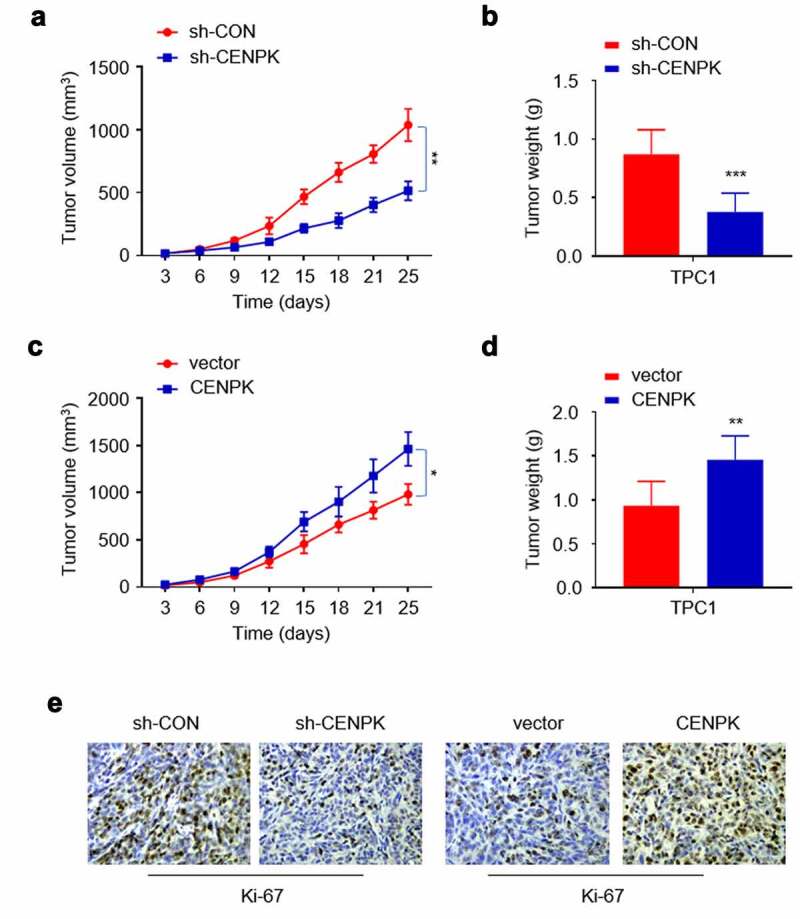
Influence of knockdown and overexpression of CENPK suppresses DTC tumor growth in vivo

**Notes**: (A) Silencing of CENPK significantly lowered the average tumor volume. (B) Silencing of CENPK significantly lowered the average tumor weight. (C) Overexpression of CENPK significantly elevated the average tumor volume. (D) Overexpression of CENPK significantly elevated the average tumor weight. (E) Silencing of CENP suppressed Ki-67 expression and Overexpression of CENPK promoted Ki-67 expression. *P < 0.05, **P < 0.01, ***P < 0.001.

Tumorigenicity assay was conducted to better analyze the CENPK effect on cell growth of nude mice. To this end, the negative control shRNA (sh-CON), sh-CENPK shRNA, CENPK-expression plasmid or vector plasmid were injected into four groups of nude mice separately. After 24 days, Ki-67 expression, tumor weight, and tumor volume decreased in shCENPK group relative to sh-CON group, while those in the CENPK group increased relative to the vector group (Figure 5a–F). The above findings suggested that CENPK silencing and CENPK overexpression may promote the DTC tumor growth in vivo.

## Discussion

The present work suggested that CENPK expression markedly increased within DTC tissue samples obtained from TCGA database, and its upregulation was evidently associated with clinical diagnosis of DTC. In addition, higher CENPK expression was found with RT-qPCR and Western blot assays in DTC tissues than in adjacent normal thyroid tissues collected in our clinical department. Therefore, CENPK possibly plays a role of the tumor-promoting molecule within DTC. According to evidence in our study, detecting CENPK may improve diagnostic accuracy, and CENPK is possibly the candidate anti-DTC therapeutic target.

Kinetochore is the great protein complex aggregated in the chromosomal centromeric region, which plays an important role in facilitating appropriate chromosomal segregation in the processes of cell growth and division [22,23]. A large amount of abnormal expression of kinetochores in tumorigenesis has been observed in multiple tumors [13,23,24]. CENPK is an important component of kinetochores, which probably generates the coiled-coils with CENP-H within the kinetochore [25]. The gene of CENPK located on chromosome 5 in humans [26]. The CENP-H/I/K complex plays an essential role in targeting CENP-A in kinetochores [15]. Additionally, KNL1 and CENP-H/I/K complex work together to bind additional CENPs to kinetochores, since co-depleting KNL1 and CENPK are found to interfere with the Hec1/Ndc80 complex’s centromeric localization [16]. Recently, CENPK expression is reported to increase in several cancer tissues in vivo and in vitro, which is related to pathological stage, T stage and histological grade, indicating the vital part of CENPK in human cancer pathogenesis [17–19]. These findings suggested that CENPK possibly exerted a vital part in kinetochore function and assembly during the cell cycle. Nonetheless, it remains largely unknown about whether CENPK affects the malignant behaviors of DTC cells and whether CENPK can be used as a biomarker of DTC diagnosis. To our knowledge, there is still no report about CENPK causing tumorigenesis and/or progression of DTC.

CENPK over-expression within DTC tissue samples reveals its potential part in the carcinogenesis of DTC. To verify this hypothesis, we conducted various experiments in vivo and in vitro. As expected, knockdown of CENPK in both TPC1 cells and FTC-133 cells markedly suppressed cell proliferation, invasion and migration in vitro, while upregulation of CENPK showed opposite affection. Besides, tumorigenicity analysis revealed that CENPK knockdown markedly inhibited cancer development while overexpressed CENPK evidently promoted tumor growth in vivo. Overall, our results support the view that CENPK may be an essential pro-tumor molecule in DTC.

The underlying mechanism for the role of CENPK in tumorigenesis of DTC is still generally unclear. Previous researches promoted several potential pathways by which CENPK may be involved in tumor proliferation, migration, and invasion. Previous research showed that CENPK might take part in regulation of many genes involved in the genesis and progression of malignant solid tumors. A recent research found that CENPK was associated with cyclinB1, an important protein related to cell cycle. CyclinB1 activation exerts a vital part in initiating the progression from G2 phase to mitosis, whereas CENPK knockdown arrests cell cycles at G2/M phase [27]. CyclinB1 over-expression is found in numerous cancers, such as breast cancer, lung cancer, and gastrointestinal tumors [28–31]. CyclinB1 was also reported to be associated with the process of proliferation and apoptosis of thyroid carcinoma cells; however, further molecular mechanism is still absent [29]. CENPK–YAP1–EMT axis is also a potential pathway taking part in biological behavior of thyroid carcinoma. Wang JL and coworkers suggested that the CENPK might participate in regulating malignant progression of hepatocellular carcinoma by CENPK–YAP1–EMT axis [18]. Yes-associated protein (YAP1) is a direct effector in the downstream Hippo pathway, which regulates numerous protein targets for regulating gene level, cell contact, proliferation, or apoptosis [32]. Recent evidence indicated that PTC tumor spheres transfected with siRNA targeting YAP1 had less differentiation potential [33]. However, whether CENPK–YAP1–EMT axis is involved in the tumorigenesis of DTC needs further exploration. Wang HY and coworkers found that overexpression of CENPK might regulate cell proliferation through AKT/TP53 signaling pathway. TP53 alteration was found to be the most frequent mutation in an anaplastic thyroid carcinoma cohort [34]. Intensive study about relationship between CENPK and AKT/TP53 signaling pathway is still absent. Overall, the association of CENPK level with the clinicopathological characteristics of DTC patients should be further investigated.

## Conclusion

This study proves that CENPK is evidently upregulated in DTC. The knockdown of CENPK inhibited proliferation, migration, and invasion of TPC1 and FTC-133 cells. Targeting the CENPK may be a new and potential therapeutic method for DTC.

## Highlights

- CENPK may play a key role in promoting carcinogenesis.
- CENPK is evidently upregulated in differentiated thyroid carcinoma (DTC).
- The knockdown of CENPK downregulates the malignant biological properties of TPC1 cells.
- Targeting the CENPK may be a novel therapeutic method for DTC.
